# Memory Deficits for Health Information Provided Through a Telehealth Video Conferencing System

**DOI:** 10.3389/fpsyg.2021.604074

**Published:** 2021-03-24

**Authors:** Benjamin Rich Zendel, Bethany Victoria Power, Roberta Maria DiDonato, Veronica Margaret Moore Hutchings

**Affiliations:** ^1^Faculty of Medicine, Memorial University of Newfoundland, St. John's, NL, Canada; ^2^Aging Research Centre-Newfoundland and Labrador, Memorial University, Corner Brook, NL, Canada

**Keywords:** telehealth, memory, hearing, healthcare delivery, aging

## Abstract

It is critical to remember details about meetings with healthcare providers. Forgetting could result in inadequate knowledge about ones' health, non-adherence with treatments, and poorer health outcomes. Hearing the health care provider plays a crucial role in consolidating information for recall. The recent COVID-19 pandemic has meant a rapid transition to videoconference-based medicine, here described as telehealth. When using telehealth speech must be filtered and compressed, and research has shown that degraded speech is more challenging to remember. Here we present preliminary results from a study that compared memory for health information provided in-person to telehealth. The data collection for this study was stopped due to the pandemic, but the preliminary results are interesting because the pandemic forced a rapid transition to telehealth. To examine a potential memory deficit for health information provided through telehealth, we presented older and younger adults with instructions on how to use two medical devices. One set of instructions was presented in-person, and the other through telehealth. Participants were asked to recall the instructions immediately after the session, and again after a 1-week delay. Overall, the number of details recalled was significantly lower when instructions were provided by telehealth, both immediately after the session and after a 1-week delay. It is likely that a mix of technological and communication strategies by the healthcare provider could reduce this telehealth memory deficit. Given the rapid transition to telehealth due to COVID-19, highlighting this deficit and providing potential solutions are timely and of utmost importance.

## Introduction

In early 2020 many countries introduced physical distancing protocols to limit the spread of COVID-19. Health authorities around the world encouraged health care providers to move to virtual care when possible. In many cases, this meant using telehealth, where a health care provider meets with a patient using voice alone (i.e., telephone) or voice and video (i.e., video conferencing). For the purposes of this paper, telehealth will refer exclusively to video conferencing between a patient and a health care provider. The rapid nature of this transition left many health care providers without training on how to deliver health care through a video conferencing system, and without supports to make this transition work. While this transition was occurring, our lab was conducting a study on how hearing status and age impact memory for health information provided through a telehealth system. Due to the COVID-19 pandemic, data collection for this study was stopped with fewer than half of the total number of participants we had planned to collect. A preliminary analysis of the data revealed a significant memory deficit for health information provided through a telehealth system compared to when the same information was provided in-person. Given the rapid transition to telehealth due to COVID-19, we felt that this preliminary data would be of interest. The next section will briefly summarize the background for the original study.

Difficulties with hearing are one of the most commonly reported health issues in older adults (Gates and Mills, 2005). Hearing difficulties make understanding speech more difficult, particularly when there is background noise, or when the speech is degraded. Interestingly, even when older adults fully comprehend mildly degraded speech, there are long-term memory deficits for the content of that speech (Pichora-Fuller et al., 1995). The proposed reason for this deficit is that a limited amount of cognitive resources that can be used at any given time, and when speech is degraded, even mildly, additional cognitive resources are needed to comprehend the speech (Pichora-Fuller et al., 1995; Schneider et al., 2010). This reduces the cognitive resources available to encode information into long-term memory (Pichora-Fuller et al., 1995; Schneider et al., 2010). Recently a series of studies demonstrated that the memory for health instructions was improved when the speech quality was enhanced, and memory for the same information was reduced when the speech was degraded (DiDonato, 2014; DiDonato and Surprenant, 2015). In the same series of studies, older adults with hearing loss benefited most when the speech was enhanced (DiDonato, 2014; DiDonato and Surprenant, 2015). This series of studies supports the idea that increased listening effort reduces memory, and demonstrates that this memory deficit occurs specifically for health information. Thus, even when older adults understand mildly degraded speech, they have more difficulty remembering what was said compared to younger adults.

One situation where this memory deficit could have major implications for older adults is for users of telehealth. Using this technology may have a significant impact on older adults, because video-conferencing systems rely on audio-compression algorithms that distort the audio signal so that information can be transmitted efficiently over the internet. The amount of distortion in the audio signal is usually dependant on the overall network bandwidth available, the quality of the microphone encoding the audio signal, and the quality of the speaker reproducing the audio signal. Proprietary digital compression algorithms can reduce the bandwidth needed to transmit the audio signal by down sampling the digital encoding and reducing the bit depth through amplitude compression. These proprietary compression algorithms, along with non-audiological grade microphones, speakers, or earphones, and non-ideal room acoustics degrade the speech. Degraded speech may not reduce the ability for an older patient to understand the healthcare provider during the session, because older adults will use additional cognitive resources in order to overcome hearing difficulties. The real problem may emerge hours or days later, when an older adult tries to remember what was said during the telehealth session. This memory deficit likely occurs because the use of additional cognitive resources to aid comprehension takes cognitive resources away from the memory system (Pichora-Fuller et al., 1995; Schneider et al., 2010; DiDonato, 2014). A recent study confirmed that older adult users of telehealth with hearing loss report more difficulty remembering what was said during a telehealth session compared to an in-person session (Willoughby and Zendel, 2017).

The goal of the original study was to experimentally test the hypothesis that older adults, particularly those with hearing loss, would have more difficulty remembering health information presented through telehealth compared to in-person after a 1-week delay. Before the COVID-19 pandemic, identifying potential memory deficits for health information presented through telehealth was critical for at least two reasons. First, telehealth use is increasing. In Canada there was a 54.6% increase in telehealth use between 2010 and 2012 (Canada's Health Informatics Association, 2013). Second, Canada's population is aging. Between 2006 and 2011, there were 1.1% more Canadians over the age of 65 (Statistics Canada, 2011). Most critical, the number of Canadians over 65, living in rural areas far from urban centres, increased 50% more than the national average between 2006 and 2011. Aging rural populations will put additional stress onto telehealth systems across Canada because older adults are more frequent users of healthcare. In 2011, older adults made up 14.8% of the Canadian population, but accounted for 45% of healthcare expenditures (Canadian Institute for Health Information, 2011). Moreover, health outcomes for older adults are more positive when they have an increased sense of control over their day-to-day lives, and are not forced to move to new communities or into long-term care facilities (Rodin, 1986). This supports the idea that aging at home benefits the health of older adults. For older adults whose home is in a remote community, this means increased reliance on telehealth. With physical distancing in place for COVID-19, identifying issues that could impact digital healthcare delivery is of utmost importance.

Unfortunately, this research was interrupted by the COVID-19 pandemic, and data collection is not complete. During the research stoppage, we explored the data and found a significant memory deficit for health information when the health information was presented through telehealth compared to in-person across all participants. Given that healthcare for many is likely to be delivered remotely for the near future, we thought these preliminary findings should be presented. Once data collection can continue, we plan to complete the study and publish the full results.

## Method

### Participants

To date, 27 participants have been recruited into the study. Ten of these participants were Younger [*M*_age_ = 27.1 (*SD* = 5.9), range 20–37; 6 female], and 17 of these participants were Older [*M*_age_ = 67.4 (*SD* = 8.8) range 51–81; 10 female]. All participants were native English speakers, and self-reported good health. All participants completed the Montreal Cognitive Assessment (MOCA; Nasreddine et al., 2005) and scored 23 or above (*M* = 28, *SD* = 2.17), the revised cut-off for mild cognitive impairment (Carson et al., 2018). On the MOCA there are two subtests associated with verbal recall: *Verbal Fluency* and *Delayed Recall*. On the Verbal Fluency participants generated 19.2 (*SD* = 5.5) words that start with the letter F, and on the Delayed Recall, participants recalled 3.5 (*SD* = 1.3) out of five words. For the full study we plan to test 60 participants (20 younger, 40 older), based on a power analysis that assumed a small-medium effect size of 0.15, an alpha of 0.05 and beta of 0.8. We planned to test a larger sample of older adults so the group could be split based on their audiological thresholds.

### Stimuli and Task

For the purpose of this brief report, only preliminary results from the experimental task will be reported. Participants also completed other audiometric and cognitive assessments, and a questionnaire about their hearing, memory, and education. These data will be reported in the final analysis. This study was approved by the Health Research Ethics Board (HREB) in Newfoundland and Labrador, and all participants provided written informed consent prior to participating. The study took part across two sessions that were 1 week apart from each other.

#### Session 1-Encoding

All testing took place in 2 adjacent teleconferencing rooms that each included a large table, and a Polycom teleconferencing system located in the Health Sciences Centre in St. John's, Newfoundland and Labrador. This type of teleconferencing system is commonly used by telehealth services in Newfoundland and Labrador. Each participant was presented with two vignettes about how to use two different medical devices (inhaler; medipatch) adapted from DiDonato and Surprenant (2015; see below for more information). One was presented in-person and the other via telehealth. In order to minimize potential practice effects, the order of presentation (telehealth or in-person, and inhaler or medipatch) was fully counter balanced between participants. Before the experimental tasks, participants completed a written informed consent, and a demographics questionnaire administered orally. Participants were instructed that they would be asked to recall the instructions of both vignettes immediately after they were presented, and again in 1 week, at the beginning of Session 2. Participants were further instructed that no information from the vignettes would be repeated, not to ask questions during the presentation of the vignette but to otherwise behave as they would during a visit with a health-care provider.

##### In-person

For the in-person condition, the participant and researcher were both seated facing each other across a conference table, about 1 meter apart. The participant could see the researchers face, arms, hands, and upper torso. The telehealth screen was off during this session to avoid distractions. The researcher read aloud the vignette about how to use one of the medical devices. The researcher spoke slowly and clearly, at a typical conversational sound level, ~60–65 dB SPL for the listener. Before testing any participants, the researcher practiced speaking at this level, using a portable sound level meter placed where a participants' ears would be to ensure they could maintain a constant level. Immediately following the vignette, participants were asked to verbally recall as much detail as they could. During recall, participants were not assisted by the researcher, and were not provided with any feedback about their accuracy.

##### Telehealth

For a vignette conducted via telehealth, the researcher moved into the adjacent room, that was nearly identical to the room the participant was in. During this session, the teleconferencing system was turned on. The teleconferencing system was a Polycom HDX6000. This type of system is used throughout Newfoundland and Labrador for telehealth sessions. Video was presented at a resolution of 1080p at 30 frames per second, and audio was presented at a 22 kHz sampling rate. The participant was seated approximately 1 meter from the screen, and 0.5 meters from the free-field speaker. The participant was able to see the researchers face, arms, hands, and upper torso on the screen. The researcher was positioned so that their image was approximately “life size” on the screen. The volume on the speaker was adjusted so that the researcher's voice was ~60–65 dB SPL where the participant was sitting. Other than being presented through the telehealth system, the task was identical to the in-person task.

##### Medical Device Vignettes

The medical device vignettes were adapted from DiDonato and Surprenant (2015). One vignette featured information on how to use a medipatch to deliver pain medication, and the other vignette featured information on how to use an asthma puffer. The vignettes were matched on many linguistic and non-linguistic aspects of speech to equate them as much as possible on the complexity of the stimuli, while at the same time maintaining their ecological validity (see DiDonato and Surprenant, 2015 for more details). Both vignettes were 10 sentences long, and included 37 details; the medipatch vignette was 154 words long, and the asthma puffer vignette was 151 words long. Reading the vignette took ~70 s, for an average speaking rate of about 2.2 words per second, which was slower and easier to understand than conversational speech (Baker and Bradlow, 2009). No visual aids or demonstrations of how to use the medipatch or asthma inhaler were provided. The 37 details were content words within each phrase that carried the most critical meaning for the purpose of using these medical devices. Details may have been a single word, compound word, or multiple words (e.g., breathe out, out of reach, etc.). The distribution of the details throughout the vignette were arranged so that each third of the vignette had a similar number and distribution of details to recall.

##### Immediate Recall

After the presentation of the vignette, the participant was asked to recall the instructions as best they could. The researcher recorded the number of details the participant correctly recalled using a scoring sheet. There was no time restriction on how long a participant could take to recall the instructions. Details did not have to be remembered in the correct order, and each detail was worth 1 point. Participants were given a score out of 37 based on how many details they correctly recalled. This score was converted to a percentage and used as a measure of Immediate Recall.

#### Session 2

After a 7-day delay, participants returned for a second session. The focus of this session was to examine the delayed recall of the medical vignettes, and to collect audiometric data. This session took place in the Cognitive Aging and Auditory Neuroscience Laboratory, a large, quiet room that contains a sound-attenuating booth, and equipment for audiometric assessments. Participants were seated at a table in an office chair facing the researcher. The researcher sat across from the participant, ~1 meter away. At this point the researcher reminded the participant that they were asked to remember the instructions given for both the medipatch and the asthma puffer. Participants were asked to describe these instructions in as much detail as they could remember. The order in which they were asked to recall the vignettes was the same as in Session 1 (i.e., if in session 1 the participant heard the medipatch vignette first and the asthma puffer second, then in session 2 the participant was asked to recall the instructions for the medipatch first, and the asthma puffer second). This order was maintained regardless of the order of presentation in session 1 was in-person or telehealth first. Scoring was identical to scoring for immediate recall, and like the immediate recall session, there was no restriction on how long a participant could take to recall the instructions. Upon completion of the delayed recall task for both vignettes, participants were given a 5-min break. Participants were then given a series of auditory and cognitive assessments. For the purpose of this brief report, this data is not presented.

## Results

**Figure 1:**
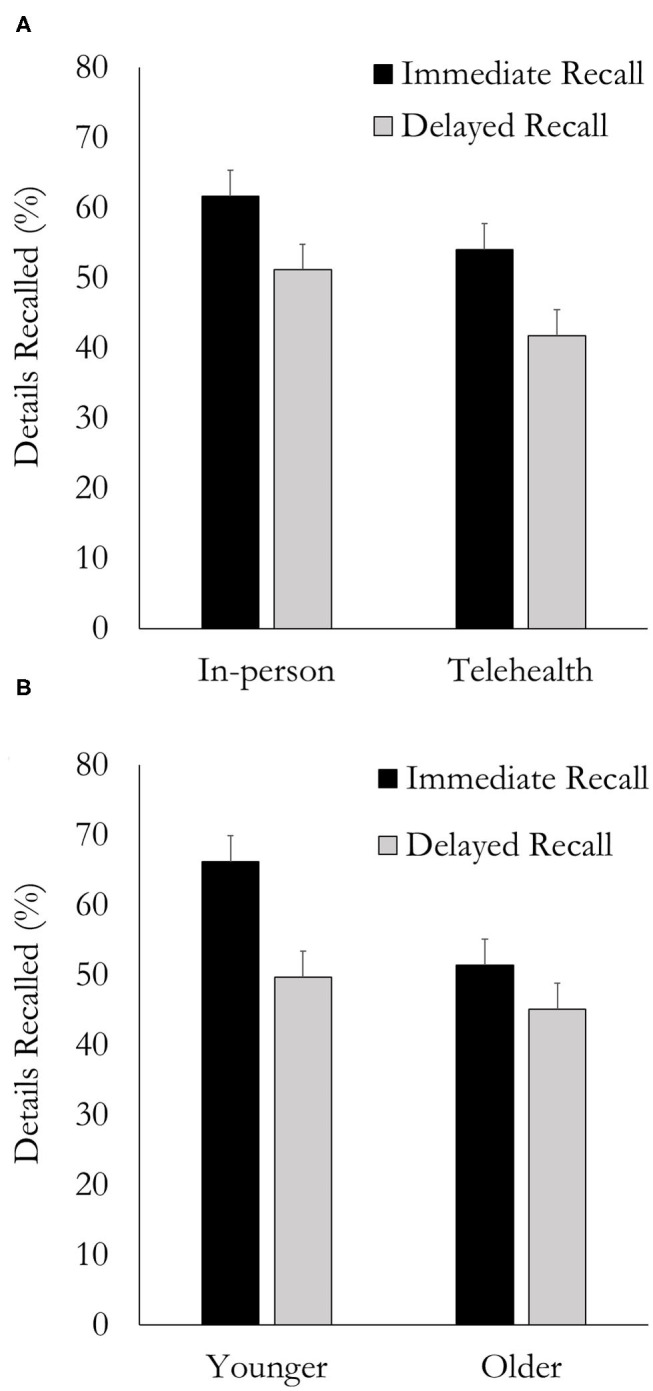
Number of details recalled immediately after the session and after a 1-week delay as a function of **(A)** Mode-of-Delivery and **(B)** Age group. Error bars are one standard error of the mean.

Data was analyzed using a mixed-design ANOVA that included Mode of Delivery (In-person, Telehealth) and Memory (Immediate, Delayed) as within-subject variables, and Age Group as a between-subject variable. The Order of presentation (i.e., Telehealth or In-person first) was initially included as a factor, but its main effect (*p* = 0.99) and interactions were not significant (*p* = 0.15–0.93), so it was removed from the analysis. There were main effects of Memory and Mode of Delivery. Overall, more details were recalled immediately after the session compared to after a 1-week delay [*F*_(1, 23)_ = 19.54, *p* < 0.001, η^*2*^ = 0.08] (Figure 1A). Additionally, more details were recalled when delivered In-person compared to via Telehealth [*F*_(1, 23)_ = 5.70, *p* = 0.026, η^*2*^ = 0.05] (Figure 1A). There was no main effect of Age Group [*F*_(1, 23)_ = 2.79, *p* = 0.11, η^*2*^ = 0.06], and there were no significant interactions between the three variables; however, the interaction between Memory and Age Group approached significance [*F*_(1, 23)_ = 3.90, *p* = 0.060, η^*2*^ = 0.02] (Figure 1B). Follow-up tests, using pairwise comparisons revealed that for Younger Adults, compared to older adults, there were larger differences in the number of details recalled immediately compared to the number of details recalled after the 1-week delay (*p* = 0.002). For Older Adults, differences in the number of details recalled immediately compared to after a 1-week-delay were smaller and not significant (*p* = 0.20).

## Discussion

Overall, both groups (younger and older) demonstrated a memory deficit when health information was presented via Telehealth compared to In-person. One interesting observation was that people remember about the same amount of information immediately after a Telehealth session (54.3%) compared to an In-Person session after a 1-week delay (51.6%), suggesting that the impact of getting health information through telehealth was similar to the impact of a 1-week delay in verbal memory recall (herein referred to as simply “recall”). Furthermore, the decrease in recall performance immediately after the session, and 1 week later was similar for both In-Person and Telehealth delivery (10.5 & 12.3%, respectively). This preliminary data suggests comprehension difficulties (i.e., impaired speech perception or cognitive-linguistic processes) due to the Telehealth mode of delivery led to a reduction in the number of details that were initially encoded. The telehealth memory deficit is unlikely to be related to differences in retention because the number of details “forgotten” during the 1-week delay was similar for both the In-person and Telehealth conditions. Once the full data set is collected a detailed exploration of the impact of aging and hearing status on immediate and delayed recall for health information provided either through telehealth or in-person will be possible.

Differences between Telehealth and In-person mode-of-delivery led to an immediate recall deficit in the Telehealth condition. The main goal of this study was to compare immediate- and delayed-recall for health information presented in two realistic situations. The main challenge was that by using a real telehealth system, we were unable to control or manipulate a number of factors that could reduce both audio and video fidelity in the Telehealth condition. The deficit we observed could therefore be related to reduced audio fidelity, reduced video fidelity or a combination of both. In a review, Mattys et al. (2012) describes this as a form of environmental/transmission degradation in communication. Mattys et al. (2012) highlights that this type of degradation could reduce speech recognition, reduce the ability to attend to the telehealth session, and reduce memory for information from the session.

There are a number of theories that can be used to interpret these results in terms of the reduced quality of the message, including The *Ease of Language Understanding* theory (Rönnberg et al., 2010), and the *Effortful Listening* theory, first introduced by Rabbitt (1968, 1991), and more recently developed into the *Framework for Understanding Effortful Listening (FUEL)* by Pichora-Fuller et al. (2016). These theories state that working memory load will increase when there is a difficulty matching incoming speech to ones' mental lexicon. This increased working memory load during listening inhibits the ability to encode details into long-term memory. Findings from the current study suggest that audio and video degradation in a commercial grade telehealth system increases working memory processing in order to accurately match incoming speech to the mental lexicon (i.e., comprehension of the message). In turn this reduced the number of details that were encoded into long-term memory, which led to a deficit in recalling health information immediately after the encoding session was complete.

Based on perceptual factors alone, one might not predict significant recall deficits due to the high quality of the telehealth system used in the study; however, a minimally degraded message can act in an insidious way. When communication is degraded minimally, an individual may not recognize the degradation, and may not engage compensation mechanisms to properly attend to, and remember what was said (Bäckman and Dixon, 1992). It is this lack of awareness that a communication event is sub-optimal that then interferes with the automatic or explicit use of those to-be-employed compensations for mitigating the effects of the degraded message (Bäckman and Dixon, 1992). In a recent review of best practices for tele-mental-health Hilty et al. (2019), specifically highlight the importance of minimizing distractions and optimizing speech clarity in order to improve the therapeutic relationship. Accordingly, in this situation, the slight imperfections in the communication associated with the Telehealth condition may have contributed to difficulty remembering what was said due to both the reduced quality of the message, and a lack of awareness of the reduced quality of the message. Previous work in visual perception has shown that target degradation increases distractor effects (Lavie and De Fockert, 2003). In the current study, this effect might be amplified because the telehealth system used was high quality, and thus the participant may not have engaged possible compensatory mechanisms (see: Bäckman and Dixon, 1992). The mildly degraded auditory and visual information in the Telehealth condition may have made minor, unavoidable distractions more salient for the individual compared to the In-Person condition. In-turn this would have reduced the number of details that were comprehended and then encoded into long-term memory.

Interestingly, this distractibility hypothesis and the Effortful Listening hypotheses make somewhat different predictions about comprehension. The Effortful Listening hypotheses predicts that immediate comprehension may indeed be similar for In-Person and Telehealth sessions with the impact of the degradation only demonstrated later as reduced recall of the message, while the distractibility hypothesis would predict that immediate comprehension may be reduced for the Telehealth session compared to the In-Person session. Unfortunately, due to the ecological nature of the study, comprehension of each statement was not measured; immediate recall was measured at the end of the vignette. Overall, it is likely that both increased distractibility and reduced fidelity of the signal play a role in reduced immediate recall. It is therefore likely that improving the fidelity of the telehealth signal, could improve memory due to reduced cognitive load, and a reduction of distractibility. Unfortunately, the current study was not designed to tease apart contributions of effortful listening and distractibility on immediate recall. This should be explored in future research.

Laboratory-based studies have shown that modifying the fidelity of speech impacts both comprehension and memory. Degraded speech has been shown to negatively impact recall compared to normal speech, including when speech is sped up (DiDonato, 2014), when it is presented with background noise (Pichora-Fuller et al., 1995) or when the speech is noise vocoded (Ward et al., 2016). Speech clarity can be improved by using inserted earphones instead of loudspeakers, and this difference has been shown to enhance recall (DiDonato and Surprenant, 2015). It is likely that providing the patient with high quality insert earphones could improve their memory from a telehealth session. The healthcare providers' speech patterns are also important for understanding. Speaking with normal prosody has been shown to improve recall compared to speaking with a flat prosody (Stine and Wingfield, 1987). One interesting finding was that older adults recalled more information when health information was presented slower, with shorter utterances, and more varied and higher pitched intonation (McGuire et al., 2000). While slower speaking has been shown to improve recall (Thompson, 1995), it has been shown that “self-paced” listening can further improve recall (Piquado et al., 2012). Self-paced listening is when the listener is allowed to pause the speech when they want. From a telehealth perspective this means that healthcare providers would be best to pause regularly. During these pauses recall could likely be further improved if patients are asked to repeat what the healthcare provider just said, as repetition is a well-known memory aid. Another important finding is that meaningful phrases are recalled better than random words (Stine and Wingfield, 1987; Thompson, 1995). This suggests that healthcare providers should take extra care to use colloquial speech, and to be thoughtful of their word choices so that each statement they make is understood and meaningful to the patient.

When speech is paired with a visual representation of the person speaking (i.e., video), comprehension improves, suggesting that videoconferencing is superior to telephone in situations where comprehension is critical (Grant et al., 1998; Grant and Seitz, 2000; Sommers et al., 2005). In these studies, the video was limited to the head and neck of the speaker, and the authors explain the audio-visual enhancement compared to auditory alone was due to an integration of audio and visual cues that facilitate phonetic and lexical decisions (Grant et al., 1998; Grant and Seitz, 2000; Sommers et al., 2005). In addition to the face, hand gestures have also been shown to improve speech understanding, although the benefit of seeing gestures seems to be reduced in older adults (Thompson, 1995; Hilty et al., 2019). In the current study, the researcher did not use their hands to demonstrate how to use the device, and based on previous work, this may have reduced the differences in recall between the in-person and telehealth conditions. Accordingly, it is important for users of telehealth to be able to see the face and hands of their healthcare provider clearly in order to see gesturing and other non-verbal forms of communication that occur through the hands.

### Summary

The current study found that immediate and long-term recall of health information was lower when that information was presented through Telehealth, compared to In-Person. Importantly, this telehealth memory deficit was similar for both immediate and delayed recall, which suggests that the best way to improve memory for health information provided through telemedicine is to improve a patients' immediate recall of the session. This is likely to be effective because the decline in recall performance after a 1-week delay was about the same regardless of the mode-of-delivery. One way to accomplish this would be to facilitate understanding so that more efficient memory encoding occurs. It is therefore likely that optimizing comprehension in telehealth situations would mitigate the listening effort and enhance immediate recall of health information. Improved immediate recall should in turn improve long-term recall. From a practical perspective, health care providers should be mindful that their patients may not recall the information presented through telehealth as they would in person, unless the health-care provider takes steps to enhance the immediate recall of information they present. The limitations of the suggestions presented here to minimize the memory deficit are based on in-person healthcare studies, or laboratory-based speech perception studies, thus they may not be generalizable to telehealth. However, given the dearth of studies on memory for information presented through telehealth, the recent need to rapidly transition healthcare into an online format due to COVID-19, and the relatively easy and low risk techniques that could improve memory, we suggest that healthcare providers attempt to use as many of the techniques reported above to improve memory for health information when they are conducting telemedicine and to review information that was presented in previous sessions. These preliminary results could be a very positive finding for telehealth, as they suggest that supplementing telehealth sessions with communication supports including high-quality earphones, and communication guidance for healthcare providers could eliminate the recall difference between in-person and telehealth sessions.

## Data Availability Statement

The raw data supporting the conclusions of this article will be made available by the authors, without undue reservation.

## Ethics Statement

The studies involving human participants were reviewed and approved by Health Research Ethics Board, Newfoundland and Labrador. The participants provided their written informed consent to participate in this study.

## Author Contributions

BZ: designed study, analyzed data, and wrote manuscript. BP: designed study, collected data, analyzed data, and wrote manuscript. RD: designed study and wrote manuscript. VH: analyzed data and wrote manuscript.

## Conflict of Interest

The authors declare that the research was conducted in the absence of any commercial or financial relationships that could be construed as a potential conflict of interest.
